# *In Vitro* Study of Degradation and Cytocompatibility of Ceramics/PLA Composite Coating on Pure Zinc for Orthopedic Application

**DOI:** 10.3389/fbioe.2022.856986

**Published:** 2022-03-04

**Authors:** Shenghui Su, Qiangqiang Tang, Dongbin Qu

**Affiliations:** ^1^ Division of Spine Surgery, Department of Orthopaedics, Nanfang Hospital, Southern Medical University, Guangzhou, China; ^2^ School of Materials Science and Engineering, South China University of Technology, Guangzhou, China; ^3^ Department of Orthopaedic Surgery, Zengcheng Branch of Nanfang Hospital, Southern Medical University, Guangzhou, China

**Keywords:** zinc, biodegradation behavior, cytocompatibility, PLA film, pH

## Abstract

Zinc and its alloys are considered to be next-generation materials for fabricating absorbable biomedical devices. However, cytotoxicity has been reported to be associated with rapid degradation. To address these issues, a composite coating (PLA/Li-OCP) consisting of CaHPO_4_ conversion coating (Ca-P) and polylactic acid (PLA) decorated with Li-octacalcium phosphate particles was constructed on pure zinc. The immersion tests showed that the presence of Ca-P coating and PLA/Li-OCP coating on pure zinc could reduce the pH value. Compared with Ca-P coating, the introduction of the PLA/Li-OCP film on the Ca-P-coated samples could enhance the corrosion resistance, and there was one order of magnitude decrease in the corrosion current density. The cytocompatibility assay suggested that the PLA/Li-OCP coating favored the cell viability and upregulated the expression of related osteogenic-genes including RUNX2, OCN, and BMP. Therefore, the presence of the PLA/Li-OCP coating on pure zinc could effectively improve the degradation rate and cytocompatibility of pure zinc.

## Introduction

Biodegradable metals such as iron, magnesium, and zinc are steadily considered to be next-generation materials for manufacturing absorbable biomedical devices due to their good mechanical properties. Currently, magnesium and iron have been studied widely because of their low elastic modulus and excellent biocompatibility. However, one of the issues against magnesium-based alloys for their clinical application is due to their rapid degradation rate and subsequent evolution of hydrogen gas in the human body environment, whereas the degradation rate of iron is very slow (Liu et al., 2018; Kabir et al., 2021; Mo et al., 2021). In contrast, the corrosion potential of zinc is between magnesium and iron, meaning that the corrosion rate of zinc falls in between that of magnesium and iron (Tong et al., 2020). Meanwhile, bone formation is closely related to zinc. Zinc deficiency would cause growth failure, neuropathy, dystocia, hypothermia, etc. (Chabosseau & Rutter, 2016). Given this, zinc and its alloys are very suitable for clinical usage, which has gained extensive interest in 2013 after the report of a landmark research from Bowen et al. (2013). According to their study, they implanted pure zinc in the rat abdominal aorta to simulate the degradation behavior of stents and found that zinc showed ideal degradation behavior in rat blood vessels compared with magnesium and iron alloys *in vivo* and that corrosion product also had good biocompatibility. Therefore, they reported that zinc and its alloys are a promising candidate as a bioabsorbable stent in the next generation.

However, cytotoxicity has been reported to be associated with the Zn^2+^ concentration released from zinc and its alloys (Murni et al., 2015; Alves et al., 2017). For example, Ma et al. demonstrated that a certain concentration of zinc ion, approximately 6.50 ppm, could inhibit cell proliferation. Peng et al. (2021) revealed that almost few cells could stay alive when the cell was directly exposed to the zinc surface (Zhu M et al., 2019). In this case, a perceived drawback of zinc is that higher concentrations of Zn^2+^ released from zinc-based alloy would induce cytotoxicity response to different cells including osteoblast. Therefore, promoting zinc and its alloys’ transfer to clinical applications still face the key issue that the high concentration of zinc ions accumulated around zinc-based implants would deteriorate the biocompatibility of implant (Zhu M et al., 2019).

Surface modification is an impactful strategy to enhance the biocompatibility of the implant by controlling the release rate of zinc ions (Pathak & Pandey, 2020; Pezzato et al., 2020; Sheng et al., 2020; Serdechnova et al., 2021). For this purpose, many studies have been focused on the improvement of bioactivity and biodegradability *via* developing surface treatments, such as PEO coating, chemical conversion coating, electrochemical deposition coating, and polymer coating (Peng et al., 2021). Conversion coating has been widely used to enhance corrosion resistance and upgrade bioactivity and biocompatibility of magnesium and its alloys by reacting magnesium with electrolyte in the bath, which is considered to be an efficient, feasible, easily applicable cost-effective way of protecting zinc and its alloys (Hafeez et al., 2020). Moreover, the calcium and zinc phosphate conversion coatings with intrinsic bioactivity and biocompatibility are proverbially produced on many biomaterials thanks to their similar composition to carbonated apatite in natural bone tissue (Guo et al., 2020; Hafeez et al., 2020). Attempts have been conducted to produce kinds of conversion coatings on zinc and its alloys (Gao et al., 2021; Zhang et al., 2020). For example, Mo et al. (2021) fabricated a zinc phosphate (ZnP) coating with decorating alendronate on zinc to improve the degradation rate and the bio-functionality and found that the coating not only improves the release rate of zinc ions but also showed the ability with balancing osteo-functionality of anti-osteoclast and pro-osteoblast response. Zhang et al. (2020) designed and fabricated a zinc-phosphate (ZnP) coating containing graphene oxide (GO) on pure zinc to control the degradation behavior of next-generation bioabsorbable implant and found that a Zn alloy scaffold with Ca-P coating could modulate Zn2+ release rate and demonstrated that the Ca-P coating could contribute to the osteogenic differentiation of BMSCs (Zhuang et al., 2021). However, the main drawback of the conversion coatings is that there is the presence of pores and cracks on the coatings, which would cause the solution to permeate into the substrate (Su et al., 2019).

Preparing a polymer film on the conversion coatings with defects can address this issue. Ostrowski et al. (2013) sealed microcracks of the MAO coating by PLLA film. Lu et al. (2011) prepared a PLA film on the Ca-P conversion coating to propose a composite coating on AZ91alloy. Their results indicated that this composite coating could reduce the biodegradation (Kannan & Liyanaarachchi, 2013). Electrochemical tests showed that the composite coating can effectively improve the polarization resistance of AZ91. et al. modified the pure zinc with the film of poly (L-lactic acid) (PLLA). The *in vivo* study suggested that the PLLA could reduce the corrosion resistance of pure zinc (Shamoli et al., 2017). Moreover, to further improve the bioactivity of the polymer films on the conversion coatings, some studies reported that bioactive elements and matters were loaded into the polymer film to promote osteogenic differentiation. doped the Li element into the MAO coating to enhance the osteogenic differentiation. The *in vitro* study suggested that the osteogenic-related gene expression was upregulated by the Li addition (Lin et al., 2021). Galli et al. noted that the differentiation of bone marrow MSCs on the titanium was improved by the Li addition on coating *via* the wnt/β-catenin signal. González-García & Monje (2013) doped octacalcium phosphate (OCP, Ca_8_H_2_(PO_4_)_6_∙5H_2_O) into the nanofibers consisting of poly(lactic-co-glycolic acid) (PLGA)/poly(caprolactone) (PCL) *via* electrospinning, and found that the nanofibers with doping OCP could upregulate the gene expression of bone-specific markers (Wang et al., 2019). Heydari et al. fabricated a novel scaffold consisting of polycaprolactone (PCL) and OCP particles. The results showed that OCP particles favored the growth of the osteoblast (Heydari et al., 2017).

Currently, the considerable attraction with zinc mainly lies in the design and development of zinc-based alloys (Ren et al., 2019; Yin et al., 2019; Zhu S et al., 2019; Tong et al., 2020), focusing on the improvement of mechanical properties, and biocompatibility evaluation. However, there are rather few studies on improving corrosion resistance and cytocompatibility for biomedical zinc *via* surface modification. Herein, the calcium phosphate conversion coatings were produced on pure zinc, following doping with a polylactic acid (PLA) with decorating Li-OCP particles to improve the biocompatibility. The microstructure, biodegradable rate, and cytocompatibility were studied to estimate the feasibility of pure zinc with Ca-P coating for clinical application.

## Experimental Details

### Materials Preparation

A zinc bar (Zn ≥ 99.99%) was cut into disk specimens with dimensions of Φ10 × 2 mm. These samples were ground with sandpapers to 2000#, ultrasonically cleaned with deionized water, respectively, and then dried in a drying cabinet. Whereafter, the samples were exposed to an acidic calcium phosphate electrolyte consisting of 0.12 M NaH_2_PO_4_, 0.20 M CaCl_2_, and 0.10 M Na_2_EDTA·2H_2_O with the pH of 3.50 for 6 h at 100°C in autoclaves. Then, the obtained Ca-P-coated samples were washed 3 times using deionized water and dried. The Li-OCP particles were fabricated by the following method: 0.009 mol NH₄PO₃ and 0.03 mol CH₄N₂O were mixed in 300 ml of distilled water for 30 min at room temperature (named A solution), while 0.01188 mol calcium acetate and 0.00012 mol of lithium chloride were mixed in 300 ml of distilled water for 30 min at room temperature (named B solution). Subsequently, solution B was added to solution A and stirred for 5 min (solution C). Solution C was heated to 90°C in a water bath with stirring for 2 h. The Li-OCP particles were obtained after the reaction. The PLA powders and Li-OCP particles were mixed and PLA was dissolved in chloroform solvent to fabricate the PLA/Li-OCP solution. Then, the Ca-P-coated samples were dipped in PLA/Li-OCP solution and pulled out rapidly to produce the PLA/Li-OCP coating on the Ca-P-coated samples (named PLA/Li-OCP-coated sample). The weight ratio of the PLA powders and Li-OCP particles was 400:1.

### Coating Analysis

A scanning electron microscope was used to observe the morphologies of coated zinc. The chemical compositions and phase of the Ca-P coatings were analyzed by energy spectroscopy (EDS) and x-ray diffraction, with the radiation source being Cu-K α with a scanning speed of 5°. The chemical valence state of coatings was characterized by x-ray photoelectron spectroscopy (XPS, ESCALAB 250Xi, Thermo Fisher) equipped with a monochromatic Al-K α (1,486.60 eV). The C1s peak located at the binding energy of 284.80 eV was used for the charge correction. The wettability of coatings was analyzed by a contact angle meter.

### Immersion Test

The corrosion rate of the coated samples was studied *via* immersion test in simulated body fluid (SBF) solution at 37 ± 0.50°C for 21 days. The volume of solution to the immersed area of the surface was 1.25 ml/cm^2^. To imitate a dynamic circulation environment in the human body, the SBF solution was refreshed every 3 days. At each time point, the pH value was recorded by a pH meter. After 21 days of immersion, the immersed coated zinc was washed by deionized water and dried in air. Scanning electron microscopy was used to observe the morphology.

### Electrochemical Tests

The corrosion resistance of coated zinc was measured *via* potential polarization curve and electrochemical impedance spectroscopy (EIS) using a Princeton Model 273A electrochemical work station in SBF solution. In the electrochemical test, the pure zinc and coated zinc were used as the working electrode, platinum sheet was used as the auxiliary electrode, and saturated mercuric electrode was used as the parameter electrode. In the dynamic potential polarization test, an applied potential ranged from −0.50 V to +1.50 V relative to OCP with a scanning velocity of 1 mV/s. The frequency ranging from 105 Hz to 10–2 Hz was used applying 10 mV perturbation in the EIS test.

### Cell Viability

Pre-osteoblast cell lines MC3T3-E1 were used to analyze the cytotoxicity. The MC3T3-E1 were cultured with studied samples in Dulbecco’s modified Eagle’s medium (DMEM) containing 100 U/ml penicillin, 10% fetal bovine serum (FBS), and 100 μg/mg streptomycin in an incubator with humid 5% CO_2_. The cytotoxicity was measured using MTT according to operating instructions. The studied coated zinc and uncoated zinc were soaked in DMEM to extract the immersion at 37°C for 3 days. The cells with a density of 5× 10^4^/well were seeded in a 96-well plate for 1, 3, and 5 days, respectively. The medium was refreshed every day. According to the manufacturer’s protocol, the cell viability in terms of optical density (OD) values was obtained at a wavelength of 490 nm in a microplate reader. Meanwhile, 5×10^4^ cells/ml were seeded on samples in 24-well plates and cultured for 3 days to assess the cell morphology attached to studied samples. After that, the cells attached to the samples were rinsed with PBS solution, and then fixed on the surface of samples using 4% paraformaldehyde at 25°C for 25 min and subsequently rinsed with PBS. Thereafter, the cell microfilament was stained by 1.0% (v/v) FITC-phalloidin dye for 30 min, and then nuclei were stained by 1 mg/ml DAPI for 10 min at 37°C. The cell morphology was analyzed by a fluorescence microscope.

### Expression of Osteogenic Genes

The mRNA expression of osteogenic differentiation-related genes including RUNX2, BMP, and OCN was detected by quantitative PCR (qPCR) analysis. The cells MC3T3-E1 with a density of 5 × 10^4^/well were cultured with studied samples in DMEM in a 24-well plate for 3 and 5 days. According to operating instructions, the mRNA expression of osteogenic genes including RUNX2, BMP, and OCN was analyzed by qPCR each time.

### Statistical Analysis

The one-way ANOVA was used for statistical analysis. There is a significance between the studied groups when *p* < 0.05.

## Results

### Coating Analysis

**FIGURE 1:**
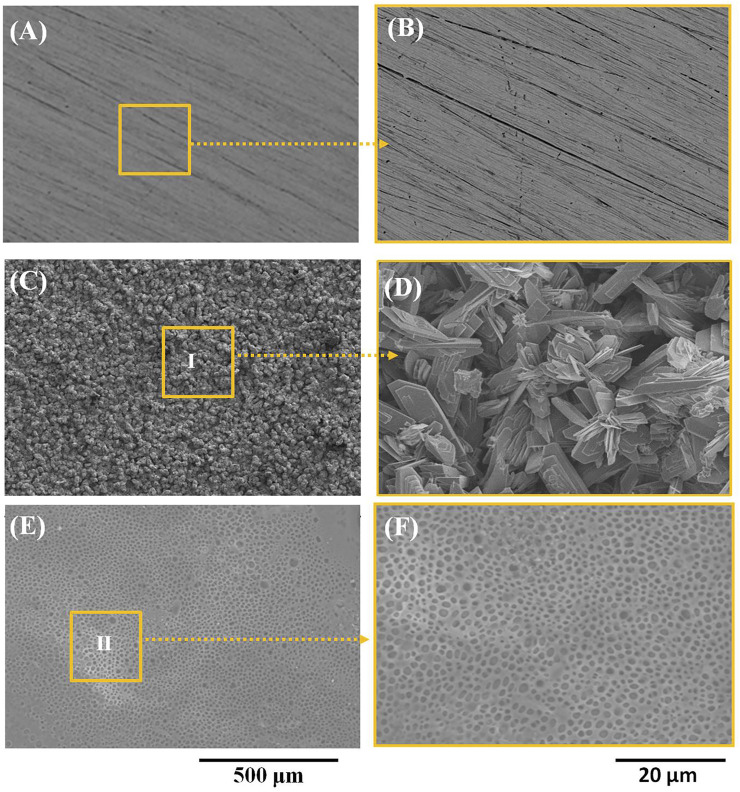
**(A, B)** The microstrucutres of pure zinc, **(C, D)** Ca-P-coated samples, and **(E, F)** PLA/Li-OCP-coated samples.

Figures 1A–F show the morphology of pure zinc, the Ca-P-coated samples, and PLA/Li-OCP-coated samples. As for the pure zinc without coating, scratches could be found on the pure zinc after being ground with sandpapers to 2000# (Figure 1A,B). With Ca-P coating treatment, at low magnification (Figure 1C), the coatings on pure zinc formed at different temperatures seem compact and dense without evident defects, indicating that the Ca-P coatings could be successfully formed on the pure zinc substrate. At high magnification (Figure 1D), numerous good crystals could be detected in all coatings, but the morphology of those coatings was different. In the case of the PLA/Li-OCP-coated samples, the morphology of Ca-P coating was covered by the PLA/Li-OCP coating. Moreover, the nano Li-OCP particles cannot be well observed in the PLA coating. The EDS results obtained from zones Ⅰ and Ⅱ are listed in Table 1, which suggested that the ratio of Ca/P for the Ca-P coating was close to 1, and the C element appeared in the PLA/Li-OCP coating, which belonged to PLA. However, the Li element cannot be detected by EDS because Li would emit an x-ray signal that exists at a relatively low intensity of less than 55 eV. This is because the conventional EDS detectors absorb 100% of x-rays of approximately 55 eV (Lin et al., 2021).

**TABLE 1:** Results of EDS obtained from zones Ⅰ and Ⅱ in Figure 1 C,E, respectively.

Element (At. %)	Ca-P coated	PLA/Li-OCP coated
O K	75.89 ± 3.88	43.95 ± 6.32
CK	n.a.	42.94 ± 8.69
P K	13.30 ± 0.26	7.19 ± 3.75
CaK	10.81 ± 0.54	4.64 ± 4.73

**FIGURE 2:**
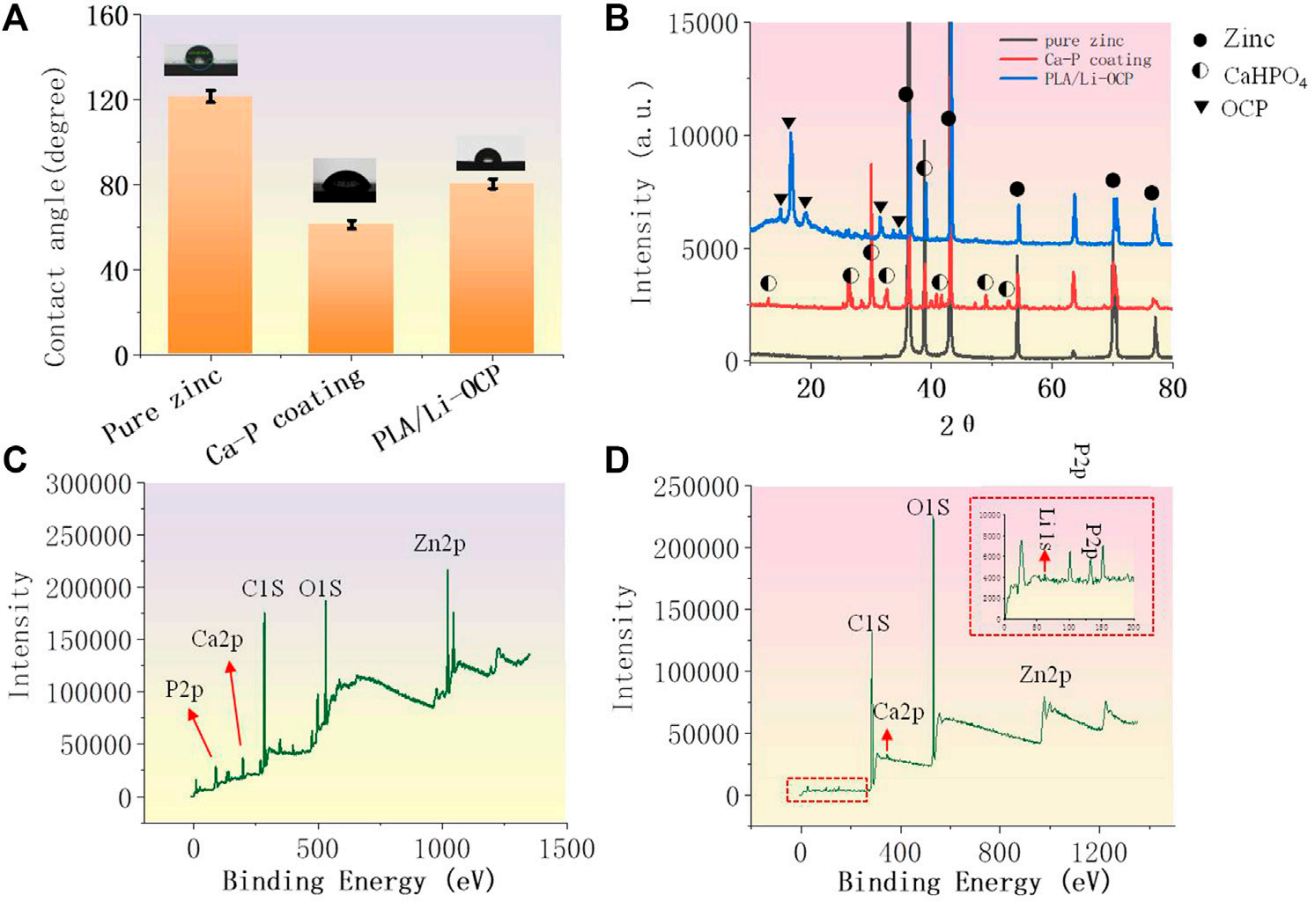
**(A)** The contact angle of studied samples; **(B)** the XRD patterns; **(C, D)** the total XPS spectrum of the Ca-P coating and PLA/Li-OCP coating.

Figure 2A shows the results of the contact angle of studied samples. Hydrophilicity is one of the indicators to assess the ability of cell adhesion on the implant in the initial stage. As can be seen, the value of contact angle of pure zinc was 121.54 ± 0.81°, and the values of contact angle for the Ca-P- and PLA/Li-OCP-coated samples were 61.12° ± 1.43 and 80.68° ± 1.05°, respectively, indicating that there was no significant difference in the water contact angle among the coatings obtained by different temperatures. Thus, the presence of coatings on the zinc could decrease the water contact angle, suggesting that the coating could improve the hydrophilicity of the surface compared to pure zinc. Figure 2B shows the XRD of the samples with and without coatings. The main phase of the Ca-P coatings was dicalcium phosphate anhydrous CaHPO_4_, whose ratio of Ca/P was consistent with the EDS results in Table 1. When the zinc was exposed to the electrolyte, heterogeneous nucleation of CaH_2_EDTA began to occur on the substrate. Meanwhile, upon processing the hydrothermal treatment, the increasing temperature promoted the precipitation of the CaH_2_EDTA crystal on the zinc surface. However, the further increasing temperature resulted in the dissolution of CaH_2_EDTA crystals, releasing calcium ions. Then, the calcium ions react with dihydrogen phosphate ions to form

$${\text{CaEDTA}}^{2-}+{\text{H}}^{+}⇌{\text{CaHEDTA}}^{-}$$ (3–1)

$${\text{CaHEDTA}}^{-}+{\text{H}}^{+}⇌{\text{CaH}}_{2}\text{EDTA}↓$$ (3–2)

$${\text{CaH}}_{2}\text{EDTA}⇌{\text{Ca}}^{2+}+{\text{H}}_{2}{\text{EDTA}}^{2-}$$ (3–3)

$${\text{Ca}}^{2+}+{\text{HPO}}_{4}^{2-}⇌{\text{CaHPO}}_{4}↓$$ (3–4)

CaHPO_4_, as a kind of biological CaP ceramics, shows excellent biocompatibility to the bone, and has been used as a coating to improve the proliferation and maturation of osteoblastic cells (Chow, 1999). As for the PLA/Li-OCP-coated samples, the main phase of the coating was Ca_8_H_2_(PO_4_)_6_∙5H_2_O. The total spectrum of the Ca-P coating in Figure 2C shows that the peaks of O1s, Zn2p, Ca2p, and P2p were detected, while Li 1s was found in PLA/Li-OCP coating except for O1s, Zn2p, Ca2p, and P2p in Figure 2D; this indicated that the Li was doped into the OCP particles.

### Electrochemical Tests

**FIGURE 3:**
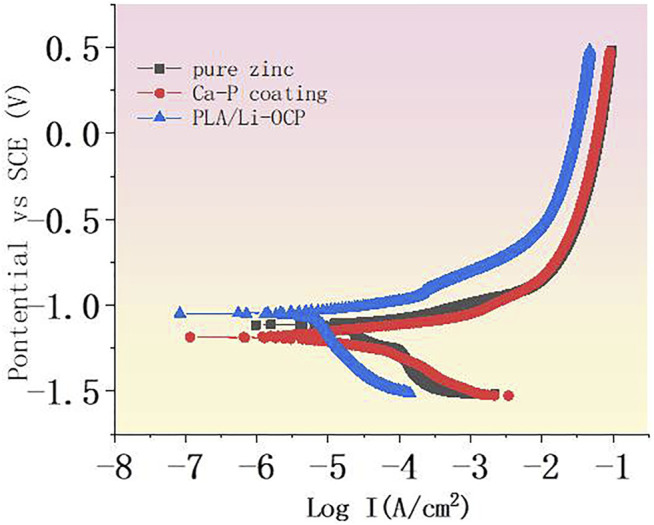
The potentiodynamic polarization curves of coated zinc and pure zinc.

The potentiodynamic polarization curves are shown in Figure 3, and the corrosion potential (*E*_*corr*_) and the corrosion current density (*I*_*corr*_) are listed in Table 2. Compared with the pure zinc, there was no significant difference in the *E*_*corr*_ between the pure zinc and Ca-P-coated samples, but the *E*_*corr*_ for the PLA/Li-OCP sample shifted to positive. The *E*_*corr*_ value of the bare zinc was −1.12 ± 0.03 V, and the *E*_*corr*_ value for the Ca-P-treated sample and PLA/Li-OCP samples was −1.18 ± 0.12 V and −1.03 ± 0.07 V, respectively. In thermodynamics, the positive shift of corrosion potential indicated that the corrosion tendency of pure zinc was inhibited by the presence of coatings on the substrate. Meanwhile, *I*_*corr*_ in the coated specimens was decreased compared with the bare zinc, in which the PLA/Li-OCP sample exhibited the lowest corrosion current density. This indicated that the PLA/Li-OCP coatings could effectively act as a barrier and block the infiltration of electrolytes into the substrate. With regard to the coated samples, the *I*_*corr*_ of the Ca-P-coated sample was reduced from 8.26 × 10^–5^ A/cm^2^ of pure zinc to 1.32 × 10^–5^ A/cm^2^. When treated with PLA/Li-OCP on the Ca-P-coated sample, the *I*_*corr*_ was further reduced to 6.56 × 10^–6^ A/cm^2^. As mentioned above, the PLA/Li-OCP-coated samples had the best corrosion resistance.

**TABLE 2:** The corresponding corrosion parameters including corrosion potential and the corrosion current density.

Sample	*I*_*corr*_ (A/cm^2^)	*E*_*corr*_ (V)
Pure zinc	(8.26 ± 0.54) × 10^–5^	−1.12 ± 0.03
Ca-P coating	(1.32 ± 0.81) × 10^–5^	−1.18 ± 0.12
PLA/Li-OCP	(6.56 ± 0.25) × 10^–6^	−1.03 ± 0.07

**FIGURE 4:**
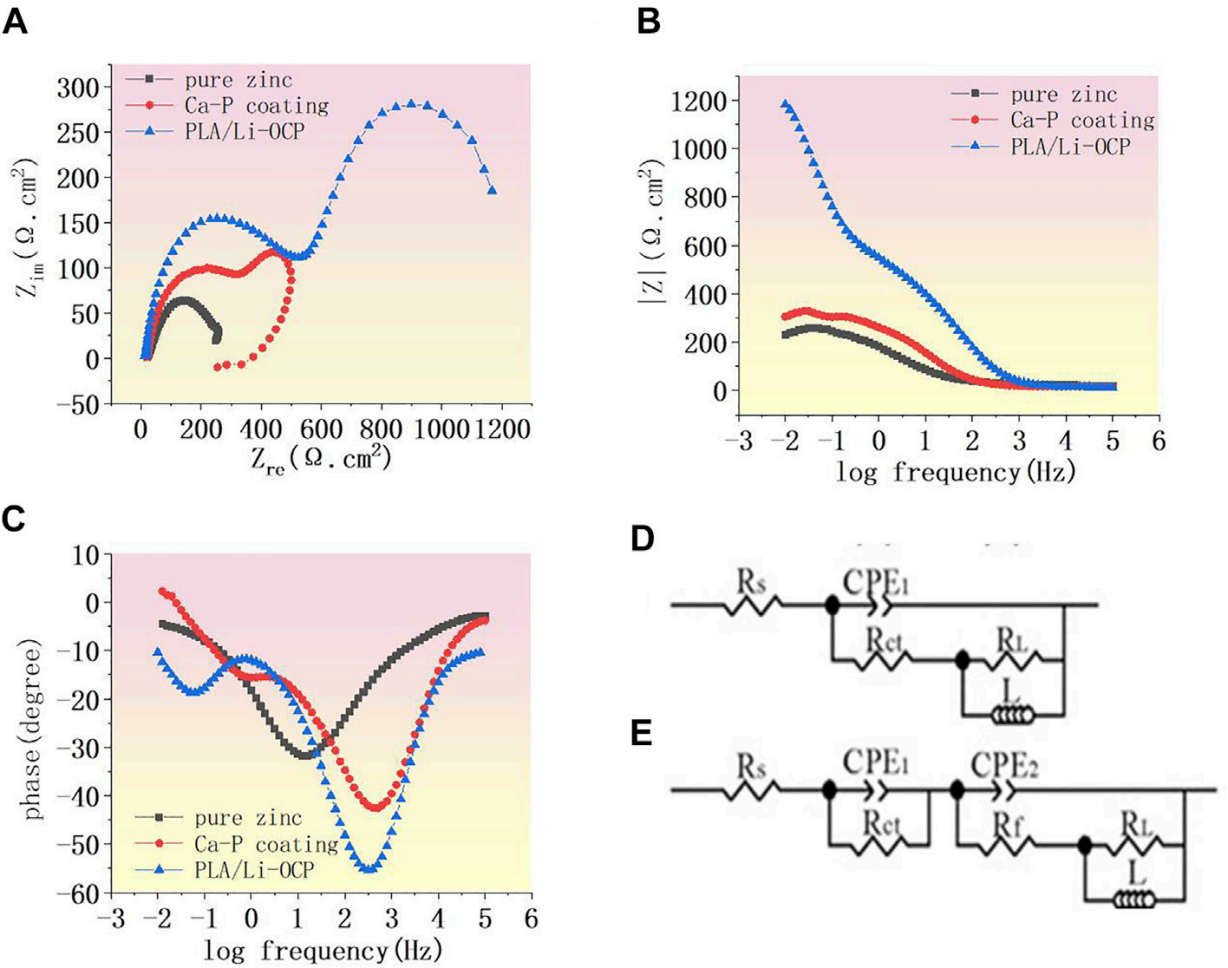
**(A)** Nyquist curves of studied samples, **(B)** Bode curves of impedance and **(C)** Bode curves of phase angle, and **(D, E)** equivalent circuit.

The EIS was used to further study the corrosion resistance of the coated samples. Figure 4A shows the Nyquist curves of studied specimens without coatings in SBF solution. In all cases, two semicircle-like curves were found in the Nyquist curves. In high frequency, the capacitive loop generally corresponds to the charge transfer resistance of corrosion products. Herein, the capacitive loop was assigned to the Ca-P coatings, while this capacitive loop in high frequency was attributed to the corrosion product for the bare zinc (Jamesh et al., 2015). The capacitive loop represented the adsorption process during corrosion. In the lower-frequency region, as a rule, the diameter of the semicircle-like curves can be represented by the polarization resistance of the coatings. In Figure 4A, the PLA/Li-OCP sample exhibited the biggest diameter of the semicircle-like curves, whereas pure zinc showed the smallest diameter of the semicircle-like curves. The Bode-impendence plots in Figure 4B show that a remarkable increase of impedance value was obtained in the coated samples compared with the bare zinc. The impedance for pure zinc, Ca-P, and PLA/Li-OCP-coated samples was ∼242.13, ∼336.65, and ∼1200 Ω cm^2^, respectively. As expected, the PLA/Li-OCP samples exhibited the greatest impedance at the lowest-frequency zone, indicating that the PLA/Li-OCP samples had the best corrosion resistance. Moreover, all the samples revealed an inductive character at the lower frequencies, a characteristic of pitting corrosion (Wu et al., 2017). The bode-phase plots in Figure 4C demonstrate that there was a significant increase in the phase angle in the medium-frequency region as the treatment temperature increased. The phase angle was reduced from −30.25° of pure zinc to −55° of the PLA/Li-OCP sample. Besides, two time constants can be found in the coated samples. Normally, in the high frequencies, the time constant is assigned to double-layer capacitance and the corresponding charge transfer resistance, while one is related to the film resistance of the corrosion product in low frequency (Wojciechowski et al., 2016). The equivalent electrical circuit was applied to fit the EIS spectra (Figures 4D,E). R_s_ means the solution resistance. CPE1 and R_ct_ are the constant phase element (CPE) and corrosion product resistance, while the constant phase element and the inner layer resistance were represented by CPE2 and R_f_, respectively (Jamesh et al., 2015). Moreover, the capacitance to remit the “scatter effect” at the interface between electrode and solution was replaced by CPE with a symbol of Q. In general, higher values of R_ct_ and R_f_ mean a greater corrosion resistance of the sample. A lower CPE1 value represents a lower corrosion area on the surface, while a higher CPE2 means the relatively thin and incompact film on the metal. The corresponding results fitted from the circuit elements are listed in Table 3, which was a good fit confirmed by chi-square (*χ* ^2^) values of about 10^–3^. The values of R_ct_ of the coated zinc for the Ca-P- and PLA/Li-OCP-coated samples were 275.34 and 275.34 Ω cm^2^, respectively, which were greater than 276.82 Ω cm^2^ of pure zinc; the values of R_f_ of the coated zinc for the Ca-P- and PLA/Li-OCP-coated samples were 25.34 and 156.47 × 10^3^ Ω cm^2^, respectively, indicating that the corrosion resistance of the inner layer was increased with temperature; besides, lower CPE1 and CPE2 values were also found in the coated zinc, indicating a near-capacitive behavior of the zinc modified by coatings. It is observed that the R_ct_ resistance of the coating was significantly larger than the R_f_ resistance of the outer layer, suggesting that the compact coating can favor more effective protection on the substrate from corrosion.

**TABLE 3:** The corresponding results fitted from the circuit element.

Samples	R_S_ (Ω.cm^2^)	R_ct_ (Ω.cm^2^)	Y0_1_ (μΩ^−1^∙sn∙cm^−2^) ×10^–5^	n1	R_f_ (Ωcm^2^) ×10^3^	Y0_2_ (μΩ^−1^∙sn∙cm^−2)^ 2 × 10^–4^	n2	R_L_ (Ω.cm^2^)	L(H) ×10^–3^
Pure zinc	20.33 ± 2.31	276.82 ± 55.87	5.22 ± 0.83	0.75 ± 0.09	—	7.022 ± 0.64	0.72 ± 0.08	257.34 ± 41.62	1.04 ± 0.36
Ca-P coating	16.95 ± 1.85	275.34 ± 42.17	3.15 ± 1.68	0.71 ± 0.13	0.025 ± 0.01	2.03 ± 0.22	0.76 ± 0.04	442.46 ± 64.21	8.52 ± 1.08
PLA/Li-OCP	18.87 ± 6.31	469.12 ± 50.64	2.74 ± 3.65	0.84 ± 0.07	156.47 ± 0.78	3.82 ± 0.98	0.89 ± 0.02	198.26 ± 24.74	0.74 ± 0.07

### Immersion Test

**FIGURE 5:**
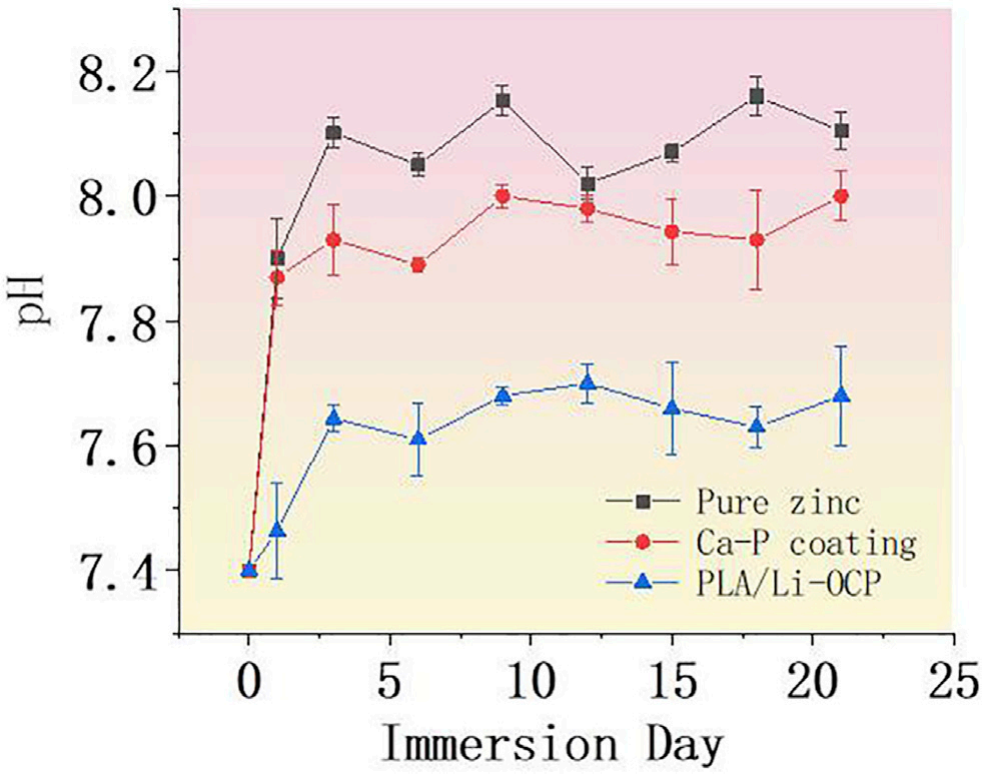
The pH value of SBF solution immersed with samples.

The pH values of SBF soaked with studied samples with and without coatings for 21 days are presented in Figure 5A. When the zinc and its alloys are exposed to the electrolyte, the chemical reaction listed as the following would have occurred (Ma et al., 2015; Hernandez-Escobar et al., 2019):

$$\text{Anodic reaction}:\text{Zn}\to {\text{ Zn}}^{2+}+2{\text{e}}^{-}$$ (3–5)

$$\text{Cathodic reaction}:{\text{H}}_{2}{\text{O}+1\text{/}2\text{O}}_{2}\to {2\text{OH}}^{-}$$ (3–6)

$$\text{Total reaction}:{\text{Zn}}^{2+}{+\;2\text{OH}}^{-}\text{Zn}\to {\left(\text{OH}\right)}_{2}$$ (3–7)

Proceeding with the anode reaction, the degradation of zinc and its alloys begins to occur. Most of the OH^−^in the cathode reaction would react with Zn^2+^ ions to form zinc hydroxide on the surface of pure zinc, while the rest of the OH^−^would cause an increase of pH value in the surrounding environment, which will promote the deposition of insoluble calcium and phosphorus salt on the zinc surface. The SBF solution immersed with the bare zinc exhibited the highest pH value approaching 7.89 on the first day, which sharply increased for 9 days, and then reduced after 12 days of immersion. With the deposition of the complex corrosion products on zinc surface, a relatively dense protective film dominantly consisting of zinc hydroxide would result in the decrease of corrosion rate. With the extension of the immersion time, the thicker corrosion layer further reduced the corrosion rate of zinc. At the same time, a degradation behavior occurred, resulting in the damage of the corrosion product layer. Then, the electrolyte infiltrated into the substrate to proceed with the degradation. This phenomenon could be reflected by the fluctuant pH curve, suggesting an alternative in pit corrosion, generation, and degradation of corrosion products on zinc. In the initial immersion time, the pH values of the SBF solutions immersed with CaP-coated samples significantly decreased, indicating that all the coatings could improve the degradation rate of pure zinc. Noticeably, it can be seen that there was a different degradation behavior among the coated samples. As for the Ca-P-coated samples, the pH value began to increase when the samples were immersed for 1 day. This suggested that some electrolyte permeated into the substrate. In contrast, the PLA/Li-OCP samples showed the lowest pH value concerning the other groups, indicating that more compact and stable coatings were deposited on pure zinc. After 12 days, the pH was gradually reduced and approached a lower level for the coated zinc. Moreover, a more fluctuant profile of the pH values in the bare zinc was observed with respect to the zinc with Ca-P coatings. With regard to coated samples, such a phenomenon was not evident in the coated groups, especially for the PLA/Li-OCP group.

**FIGURE 6:**
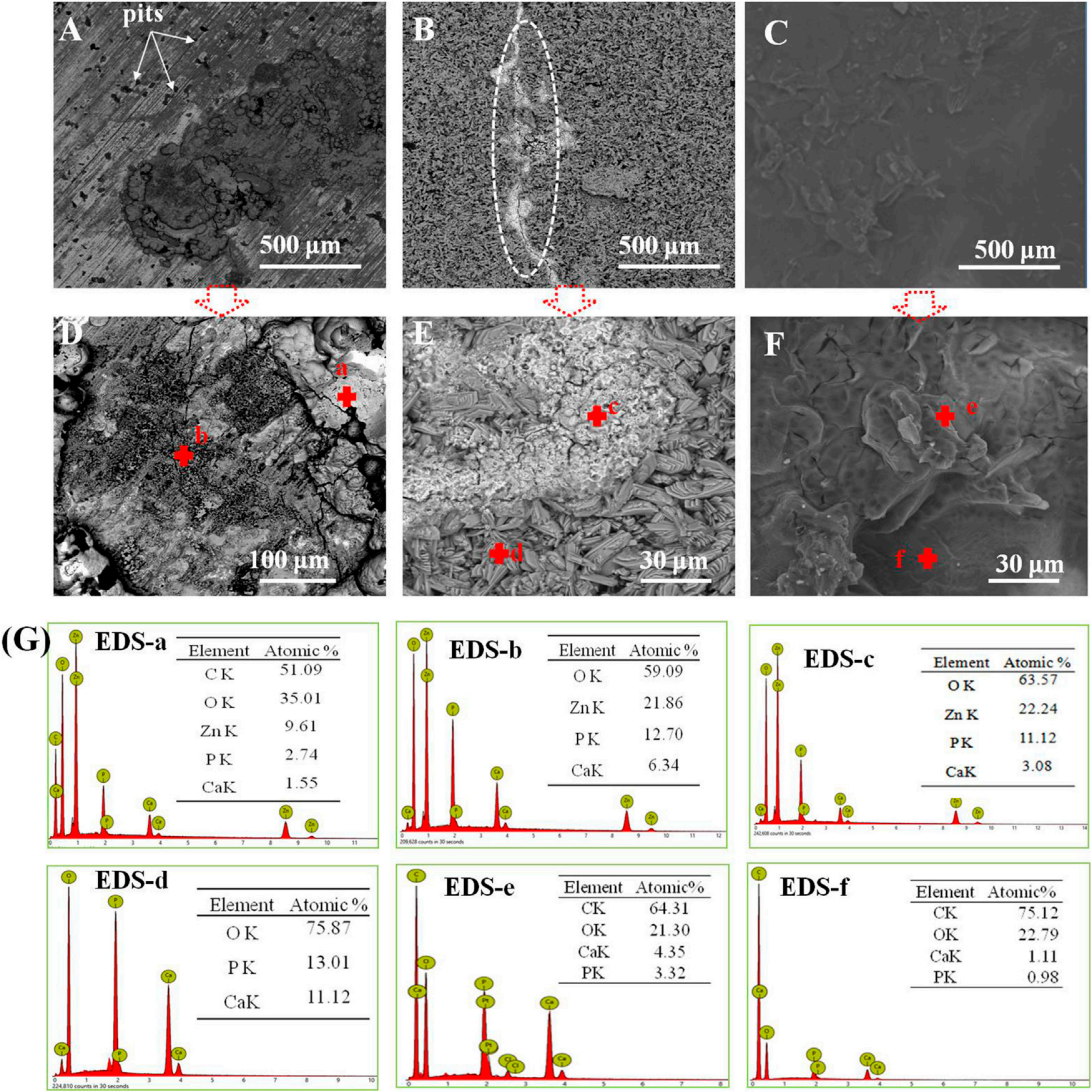
**(A–G)** The microstructures of the studied samples immersed in SBF for 21 days and the corresponding EDS patterns in panel **(G)**: **(A, D)** pure zinc; **(B, E)** Ca-P coating; **(C, F)** PLA/Li-OCP coating.

Figures 6A–G display the morphology of the studied samples immersed in SBF for 21 days, and the corresponding EDS patterns are shown in Figure 6G. It can be seen that lots of pits were displayed in pure zinc, indicating that pure zinc suffered from pitting corrosion, as seen in Figure 6A,D; moreover, the EDS patterns obtained from *point a* and *b* in Figure 6D suggested that the corrosion products consisted of Ca and P. In the case of the Ca-P-coated sample in Figure 6B, a crack could be observed on the coating, as marked by the ellipse, and the EDS patterns from *point c* in Figure 6E indicated that this region should be assigned to the corrosion products. This is because the Zn element appeared in this region compared with *point b.* The presence of Zn demonstrated that the occurrence of pit corrosion resulted in the released zinc ions from the substrate reacting with calcium phosphate to form corrosion products, suggesting that the protection of Ca-P coating locally became invalid, resulting in the electrolyte permeating into the substrate. Concerning the PLA/Li-OCP coating (Figure 6C), there was still a compact and dense PLA coating without obvious defects on the substrate. This suggested that the PLA coating could provide ample protection to prevent the electrolyte from infiltrating the substrate. This is further confirmed by the EDS patterns in *point e* in Figure 6F, which showed that no Zn element was found in the coating, and a small amount of Ca and P was found, indicating sufficient protection from the coating although there was degradation of the PLA coatings.

### Cell Activity and Osteogenesis Differentiation

**FIGURE 7:**
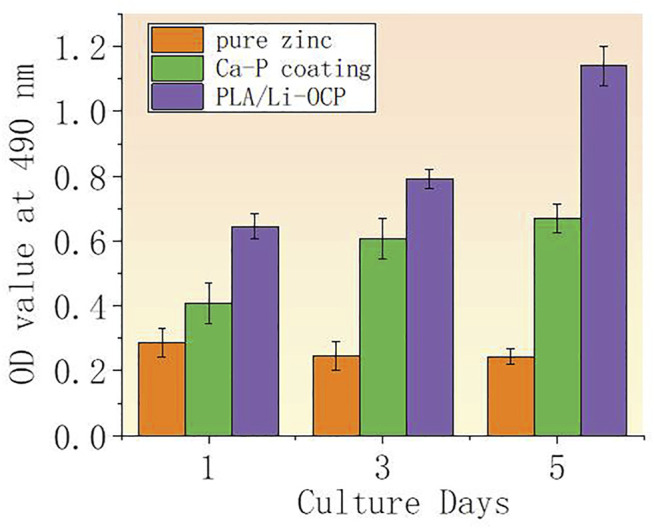
**(A)** OD value after MC3T3-E cells being seeded on studied samples for 1, 3, and 5 days (**p < 0.05).

**FIGURE 8:**
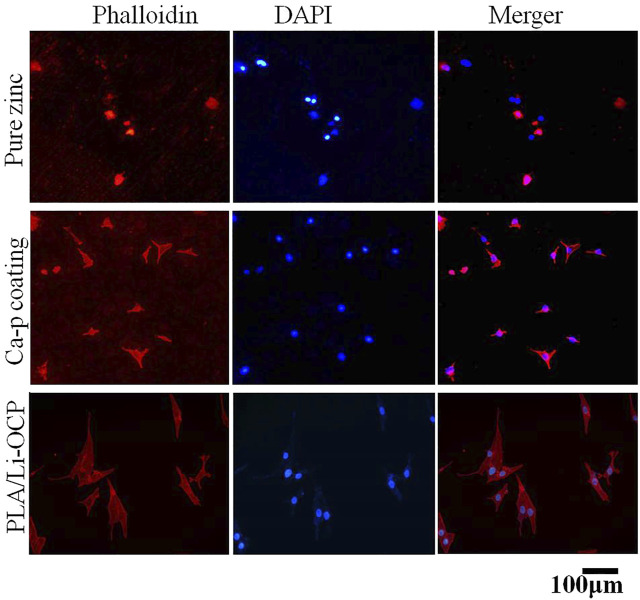
Immunofluorescence-stained osteoblasts on samples after 3 days of culture.

The cell viability after cells were seeded on studied samples with and without coatings for 1, 3, and 5 days is shown in Figure 7. It can be seen that there was almost no cell activity when the cells were exposed to the bare zinc during culture time, which suggested severe cytotoxicity of the material. This could be attributed to the high zinc ion concentration and pH value that induced cell apoptosis. In contrast, such a case was improved in that cell activity could be detected when the cells were co-cultured with the coated samples. As for the Ca-P-treated group, the maximum cell viability was found on the first day and then the value of viability slightly decreased with time. The cell viability for the PLA/Li-OCP group was higher than that of the Ca-P-treated samples at any time point, and the cell viability gradually increased after 3 and 5 days of culture. This revealed that the PLA/Li-OCP sample showed the greatest cytocompatibility with the ability for cell proliferation due to the degradable rate being considerably improved by the coating obtained at this temperature. Figure 8 shows photographs of MC3T3-E1 cells exposed on the studied samples for 3 days. In Figure 8, alive cells failed to adhere to the pure zinc surface, showing lytic morphology, a characteristic of dead cells. This case was slightly changed in the Ca-P-coated samples. In contrast, these conditions were changed by the presence of PLA/Li-OCP coatings. It can be seen that the cells attached to the PLA/Li-OCP coatings show a healthy spreading morphology, indicating that the PLA/Li-OCP coatings could favor the attachment and proliferation of cells. This observation coincided with the results of cell activity.

**FIGURE 9:**
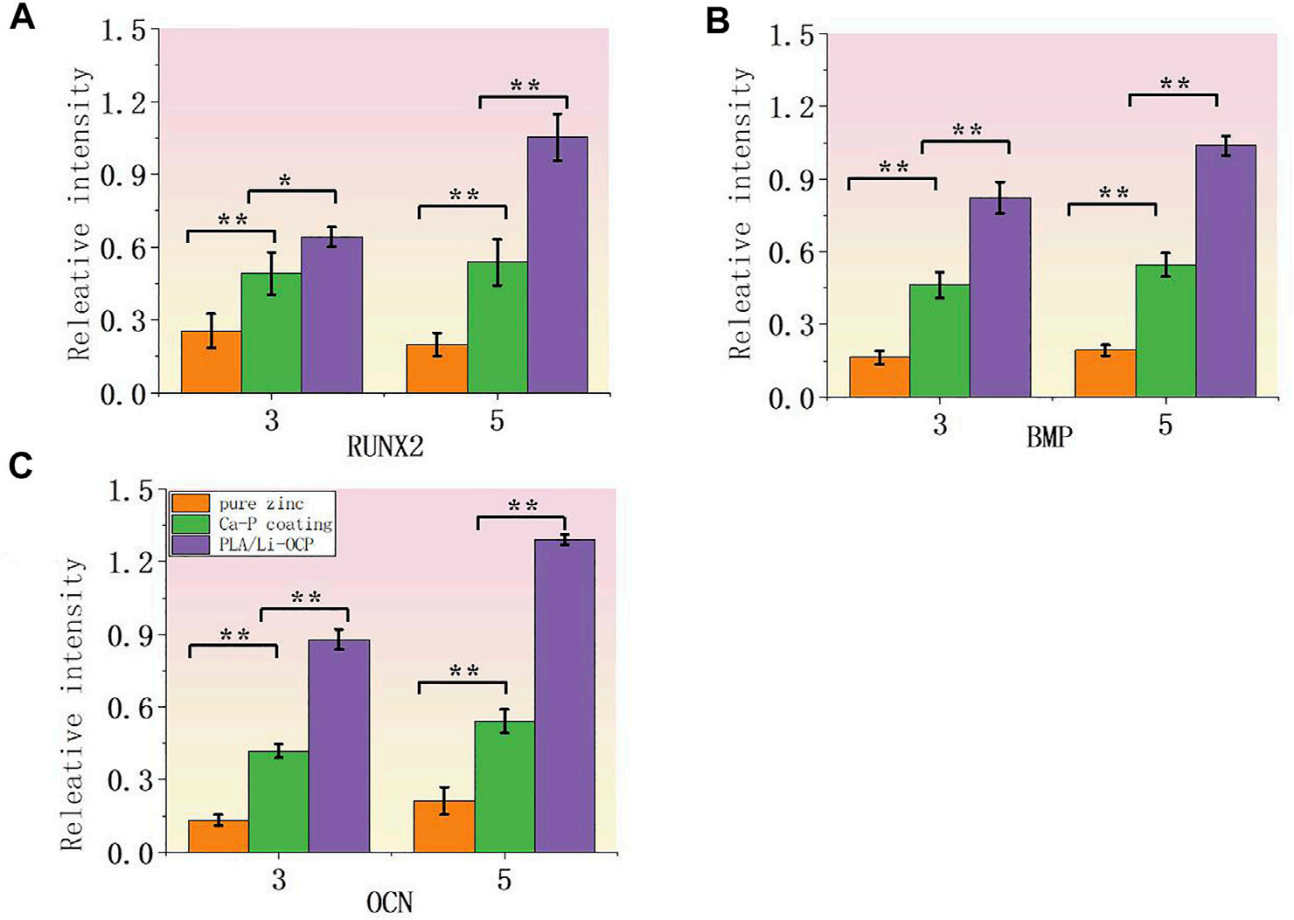
Expression of related osteogenic genes: **(A)** RUNX2, **(B)** BMP, and **(C)** OCN (*p > 0.05, **p < 0.05).

To further evaluate the effect of coating on cell proliferation, RT-PCR was applied to measure the related osteogenic genes at the mRNA level including runt-related transcription factor 2 (RUNX2), osteocalcin (OCN), and bone morphogenetic protein (BMP). Figures 9A–C show the expression levels of RUNX2, BMP, and OCN after cells were cultured with samples for 3 and 5 days. As expected, the expression of all related osteogenic genes in the bare zinc was much low during culture time, whereas fabricating the Ca-P coatings on the zinc could slightly enhance the expression of related osteogenic genes. After 3 days of culture, the expression of RUNX2 and BMP on Ca-P-treated samples was higher than that on the pure zinc, and the PLA/Li-OCP samples present the greatest gene expression among the other groups. After 5 days of culture, the expression of the osteogenic genes in PLA/Li-OCP-treated samples was higher than that of the Ca-P-treated samples and pure zinc. As mentioned above, the PLA/Li-OCP coating could improve the osteogenic activity and osteogenesis of pure zinc.

## Discussion

Once an implant is fixed in the body, cells subsequently adhere to the implant for cell proliferation and differentiation, and cell adhesion has a close relationship with the cultural environment induced by the materials. Therefore, material without cytotoxicity to the cells is a prerequisite for the healing of the bone defect. For zinc and its alloys, one of the main obstacles to transfer to clinical application is the locally accumulated zinc ions in the initial stage of the degradation process, which inhibits the cell adhesion to the materials. Although zinc is required for bone formation, there is a piece of increasing evidence showing that an excessive release of zinc ions from an implant would induce cytotoxicity. Yuan et al. (2019), who investigated the effect of zinc ions on the endothelial cellular responses, demonstrated that a low concentration of zinc ions less than 80 μM could contribute to cell adhesion and proliferation. However, when the zinc ions exceed 80 μM, a negative side to the cell viability would occur (Ma et al., 2015). According to the study from Murni et al., the high zinc ion concentration released from the Zn–3Mg alloy could inhibit electron transport in uncoupled mitochondria (Murni et al., 2015). This drawback was improved by introducing PLA/Li-OCP coating on pure zinc in this study. The important observation that aids understanding of this improvement is the decrease in pH value and releasing concentration of zinc ions. Moreover, the protective effect of coatings on the substrate was enhanced with increasing treatment temperature. As analysis from immersion and electrochemical tests shows, the PLA/Li-OCP coating exhibited the best protective effect. During immersion, herein, three stages of degradation could be proposed for the Ca-P coating, namely, coating degradation, local pit corrosion of substrate, and deposition of corrosion products. In the coating degradation stage, the coatings began to degrade when the sample was exposed to the electrolyte. The local degradation of coating provided sites for solution infiltration into the substrate, causing the local pit corrosion of substrate, accompanied by the release of OH^−^ and zinc ions. This phenomenon could be indicated by the elevated pH value and zinc ion concentration. Subsequently, a locally high concentration of zinc ions reacted with the OH^−^ and the phosphate radical to form corrosion products, which, in turn, deposited on the coatings, leading to the decrease of pH value and zinc ion concentration. In fact, the degradation behaviors of all the coatings were different in PBS solution, as the protective effect of coatings is related to microstructure and thickness (Hiromoto, Tomozawa, & Maruyama, 2013). For example, the increasing pH value in the Ca-P-treated group occurred after 5 days, which should be due to the unstable coating obtained at low temperature, and early damage of coating was present. On the contrary, the pH value of the PLA/Li-OCP group remained more stable due to the compact coatings (Pan et al., 2009). The presence of the PLA coating could further prevent the solution from permeating into the substrate.

Accordingly, the cell activity on different coatings suggested that the coated zinc showed good cell proliferation compared with the bare zinc. As such, the outcome from real-time PCR indicated that the pure zinc modified with Ca-P coating and PLA/Li-OCP coating could upregulate the osteogenic gene expression including RUNX2, OCN, and BMP. These findings indicated that the coated zinc could favor osteogenic differentiation in the early and late osteogenesis process. On the one hand, the improvement of cytocompatibility should be attributed to the decrease in local zinc ions, which provides a healthy microenvironment for cell attachment and differentiation. That is, the Ca-P and PLA/Li-OCP coating acted as a barrier to close the corrosive liquid of zinc matrix and modulated the degradation rate. This inhibitory effect promoted the generation of a friendly microenvironment for cell proliferation and differentiation, because the cell medium was refreshed every day. On the other hand, the reduced pH value also helps cell adhesion and differentiation, since cell adhesion on biomaterials is closely relevant for the alkaline microenvironment (Lin et al., 2021). In Figure 5, it can be found that the pH value of pure zinc on the first day approached 7.98, whereas the pH value of Ca-P and PLA/Li-OCP samples was 7.78 and 7.42. The decrease in pH value by the introduction of Ca-P coating was the other factor promoting cell differentiation. Noticeably, the presence of the Ca and Li elements in the coating may also be a factor to favor the enhancement of cytocompatibility. Ninety-nine percent of the Ca element in the body is present in normal bone tissue, and Ca ions can recruit a certain number of bone growth precursor cells to the damaged site by activating calmodulin signaling and activation of ERK1/2 and PI3K/Akt pathway, thereby stimulating the adhesion, proliferation, and differentiation of human osteocyte (Hu et al., 2019; Gao et al., 2021). Moreover, lithium (Li) ions, as a trace element in the human body, were considered to play an important role in organisms (Lin et al., 2021). According to Chen et al., osteogenic differentiation was found to be promoted by adding Li ions in the inflammatory microenvironment (Chen et al., 2013). In addition, it has to be mentioned that the OCP also contributed to enhancing biocompatibility. Many studies have observed that the OCP-based materials favor the bone regenerative properties *via* promoting the osteoblast differentiation from precursor cells *in vitro* (Suzuki, 2013). However, we have to mention that the promotion of cell activity favored by the presence of PLA/Li-OCP coating on pure zinc may be complex, which is the result of synergism of dynamic change of zinc ion, pH, and the existing Ca and P ion. Therefore, an in-depth investigation should be performed *in vitro* and *in vivo* in further studies.

## Conclusion

In this study, a composite coating consisting of CaHPO_4_ conversion coating (Ca-P) and PLA decorated with Li-octacalcium phosphate particles (PLA/Li-OCP) was constructed on pure zinc to improve degradation and cytocompatibility. The presence of the PLA/Li-OCP coatings elaborated an effective barrier to prevent the electrolyte from infiltrating the substrate. The enhanced corrosion resistance significantly reduced the pH value, resulting in improving the cytocompatibility. Moreover, the improvement of degradation also upregulated the expression of related osteogenic genes including RUNX2, OCN, and BMP. Therefore, the PLA/Li-OCP conversion coating was a promising coating to improve degradation rate and cytocompatibility.

## Data Availability

The original contributions presented in the study are included in the article/Supplementary Material. Further inquiries can be directed to the corresponding author.
